# Harnessing artificial intelligence in radiology to augment population health

**DOI:** 10.3389/fmedt.2023.1281500

**Published:** 2023-11-08

**Authors:** Jordan Z. T. Sim, K. N. Bhanu Prakash, Wei Min Huang, Cher Heng Tan

**Affiliations:** ^1^ Department of Diagnostic Radiology, Tan Tock Seng Hospital, Singapore, Singapore; ^2^ Clinical Data Analytics & Radiomics, Cellular Image Informatics, Bioinformatics Institute, Singapore, Singapore; ^3^ Healthcare-MedTech Division & Visual Intelligence Department, Institute for Infocomm Research, Singapore, Singapore; ^4^ Lee Kong Chian School of Medicine, Nanyang Technological University, Singapore, Singapore

**Keywords:** imaging, radiology, artificial intelligence, population health, machine learning

## Abstract

This review article serves to highlight radiological services as a major cost driver for the healthcare sector, and the potential improvements in productivity and cost savings that can be generated by incorporating artificial intelligence (AI) into the radiology workflow, referencing Singapore healthcare as an example. More specifically, we will discuss the opportunities for AI in lowering healthcare costs and supporting transformational shifts in our care model in the following domains: predictive analytics for optimising throughput and appropriate referrals, computer vision for image enhancement (to increase scanner efficiency and decrease radiation exposure) and pattern recognition (to aid human interpretation and worklist prioritisation), natural language processing and large language models for optimising reports and text data-mining. In the context of preventive health, we will discuss how AI can support population level screening for major disease burdens through opportunistic screening and democratise expertise to increase access to radiological services in primary and community care.

## 1. Background

The ageing population, chronic disease burden, and rising healthcare costs are major challenges facing healthcare systems worldwide. According to the World Health Organization (WHO), by 2050, the world's population aged 60 years and older is projected to reach 2 billion, up from 900 million in 2015 (1). As people age, they are more likely to develop chronic diseases such as diabetes, heart disease, and cancer, which require long-term management and often lead to disability, reducing quality of life and increasing healthcare costs. According to latest estimates by the Organization for Economic Cooperation and Development (OECD), healthcare spending per capita is projected to increase by an average of 2.7% annually across OECD countries between 2015 and 2060 and healthcare expenditure is set to outpace GDP growth to 2030 (2).

**Figure 1:**
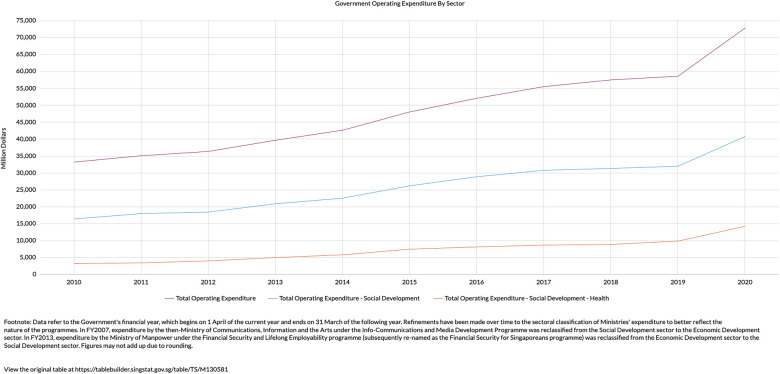
Increase in total operating expenditure (purple line) is due to a disproportionate increase in expenditure related to health (orange line) (4).

The situation in Singapore is similar, where healthcare spending is expected to form bulk of the increase in government social expenditure by 2030 (3). Government healthcare expenditure has grown exponentially in recent decades, seeing a 300% increase from the year 2010 to 2020 (Figure 1). This disproportionate increase saw healthcare expenditure taking up 18% of total government expenditure in 2020, up from just 8% in 2010 (4). In short, healthcare spending in Singapore is rising at an unsustainable rate, fostering a tremendous impetus for Healthier SG.

## 2. Healthier SG in Singapore: adopting the paradigm of population health

In line with a global shift towards Population Health, Singapore recently launched Healthier SG with focus on five key features: mobilizing family doctors to provide preventive care for residents, creating health plans that encompass lifestyle changes, regular health check-ups, and necessary vaccinations, engaging community partners to assist residents in leading healthier lifestyles, initiating a nationwide enrolment campaign for residents to commit to seeing one family doctor and following a health plan, and establishing essential support systems like IT, manpower development plans, and financing policies to ensure the success of Healthier SG (5). Overall, the greater emphasis on preventative health aims to not only better manage chronic disease burden but also reduce costs by keeping residents out of hospitals and away from high-cost medical events.

The launch of Healthier SG is in line with the Quadruple Aim of healthcare. The Quadruple Aim is a framework that was developed to guide healthcare organizations and providers in (i) improving population health, (ii) enhancing patient experience and outcomes (iii) reducing cost of care and (iv) addressing provider burnout (6).

Artificial intelligence (AI) has the potential to contribute to each of the four components; for example:

i. Improving population health: AI is essential in analysing the big data that comes with population health, identifying trends thereby allowing healthcare providers to make informed decisions about public health interventions.
ii. Improving patient experience: AI can be used to personalise and streamline the patient experience, for example via AI-powered chatbots, virtual assistants to provide immediate Q &A, and tracking individual health status and notifying the patient for living behaviour change and clinical follow-up etc.
iii. Reduced healthcare costs: AI can help to identify waste and inefficiencies in healthcare delivery, allowing for targeted cost-saving interventions. At the same time, through early warning and early discovery of health issues of each individual, the proactive intervention could dramatically reduce the overall treatment cost.
iv. Improving healthcare provider well-being: AI can automate repetitive tasks in assisting anatomic structure delineation (e.g., delineating coronary arteries on CT coronary angiography), lesion finding and measurement (e.g., following up tumour progression through serial measurements), reducing workload and freeing up more time for meaningful patient interactions. Other examples include improving the throughput for pathology reporting, radiology reporting, and expediting triage for treatment.

The limitless possibilities of AI are beyond the scope of this article. The following paragraphs will focus on radiology as a major cost driver for the healthcare sector, and the potential improvements in productivity and cost savings that can be generated by incorporating AI into the radiology workflow.

## 3. Radiology as a major cost driver

Globally, radiology services are a major drive of increasing healthcare costs. The amount that countries spend on radiological services varies widely depending on several factors such as the size of the country, population, healthcare priorities, and the level of development of the healthcare system. Although the exact expenditure by countries on radiological services is not readily available online, the increased cost can be extrapolated from the commensurate increase in use of radiological services.

A 2018 report by OECD noted a significant increase in use of radiological services over the past few decades. Between 2000 and 2016, the number of computed tomography (CT) scans and magnetic resonance imaging (MRI) scans performed in OECD countries increased by more than 60% and 80%, respectively (7). The same report highlights that the use of radiological services has increased faster than the overall growth in healthcare spending, underlining the importance of making these services more cost-effective as they become more prevalent.

To meet the rising need for advanced imaging technology, skilled personnel or tools in the diagnostic field are essential and recruitment and retaining talent adds to the increased cost. The scarcity of skilled workers can be attributed to a stagnating number of trained radiologists and radiological technicians, against a backdrop of rising demand for radiology services. To satisfy the demand for increasing cross-sectional imaging, Royal College of Radiologists (RCR) in the UK has set a target of a minimum of eight full-time equivalent (FTE) radiologists per 100,000 population. The 2022 number stands at 5.7 FTE per 100,000 population (8). In other high-income countries, the proportion of radiologists per 100,000 population varies between 4.7 (minimum) and almost 12.0 (maximum) in Sweden. The US reported 8.7 radiologists per 100,000 population in 2014 (9).

Singapore has a relatively lower workforce per capita and are still playing catch-up in our service provision to meet the needs of the population. In Singapore, the ratio of radiologists per 100,000 population was 5.2 (with a total of 286 radiologists) in 2014. This figure rose to 6.4 (with a total of 357 radiologists) in 2017 and further to 7.2 (with a total of 409 radiologists) in 2020, as reported in (10). Similarly, the ratio of radiological technicians per 100,000 population increased from 23.8 (with a total of 1,300 technicians) in 2014 to 28.1 (with a total of 1,579 technicians) in 2017 (11). While these growth rates seem promising, it is crucial to make sustained efforts to maintain a steady influx of next-generation radiologists and radiological technicians to meet future demands.

In addition to bolstering the workforce, adoption of AI into radiology workflows can also lead to improvements in productivity, at the same time delivering significant cost savings. Increased productivity via AI can be achieved in many ways and while it is challenging to quantify the cost savings for each particular use case, some surrogate markers (interpretation time, scan time, waiting time etc.) provide related information. For example, deep learning models developed in Singapore improved productivity in conventional radiography interpretation (22% reduction in turn-around time) during real-world clinical deployment (12) and even had an impact on reporting times of more advanced modalities such as MRI scans (13). An automated triaging solution in UK reduced average reporting delay from 11.2 to 2.7 days for critical imaging findings (14). A recent US-based study estimates 25% cost savings from early diagnosis of cancers (15), an achievable goal with AI assistance and/or augmentation. We are at cusp of mainstreaming AI into clinical practice. Early work has shown marginal improvements. With more widespread adoption, we can expect a more significant improvement in productivity.

The recent launch of Healthier SG will effect a transformational change in healthcare delivery in Singapore. Investing in primary prevention of diseases presents opportunities for radiological services to contribute more effectively to the health system. Although diagnostic radiology plays a vital role in healthcare, the current care model is typically centred around tertiary facilities and focuses on high-end cross-sectional imaging at late disease stages. As part of the healthcare system, radiology must shift its focus towards enhancing general population health and promoting early diagnosis and intervention. Adopting a value-based care approach is crucial to avoid escalating healthcare costs, over-testing, potential adverse patient outcomes, and overburdening limited healthcare resources (16). Healthcare of the future will be more proactive rather than reactive; radiology is not spared from this inevitable paradigm shift, and this can all be enabled by AI. The next few paragraphs will touch on the opportunities for AI in lowering healthcare costs and supporting transformational shifts in our care model.

In the next section, we discuss in greater detail how and what AI can do in the modern radiology workflow, we will also examine the key issues revolving the design and utility of AI models for them to have any significant impact.

## 4. How AI can help: opportunities for AI in the radiology workflow and lowering healthcare costs

In this section, we examine the opportunities AI present in radiological services regarding healthcare cost reduction and supporting transformational shifts in the healthcare model. In a typical radiology workflow, the clinician orders an imaging examination for the patient, scheduling occurs, and patient arrives on the examination day, possibly having done some pre-imaging preparation (e.g., fasting, stopping anti-coagulants for procedures etc.). Further patient preparation takes place (e.g., IV cannulation, checklist for metallic implants etc.) before image acquisition. The images are then processed before materializing on a worklist for radiologists to interpret and report.

With that the aforementioned in mind, the ensuing discussion will be following the radiologic imaging and diagnostic workflow: (i) Prior to image acquisition (ii) Image acquisition, processing and triage (iii) Image interpretation and reporting. Finally, we will touch on the (iv) Role of radiology in population health.

### 4.1. Prior to image acquisiton

To improve productivity, lower healthcare costs and increase efficiency, technological solutions must be deployed beyond the imaging suite. In Singapore, Changi General Hospital demonstrated a 17.2% improvement from baseline no-show rates via telephone reminders triggered by AI identification of high-risk patients (17). Defaulted radiology appointments can have significant downstream effects, delaying corresponding clinical appointments and thus treatment. Later-stage diseases are harder and costlier to address.

Apart from reducing no-show rates, inappropriate use of imaging services should also be curbed. The Agency for Care Effectiveness, Ministry of Health (MOH) in Singapore launched the Appropriateness Criteria for Use of Imaging Technology (ACUITy) project in 2018. Radiologists oversaw this project, but they also enlisted the assistance of multidisciplinary panels from other Chapters and Colleges of the Academy of Medicine, Singapore (AMS). Today, three ACE Clinical Guidances (ACGs) spanning several imaging modalities and numerous clinical scenarios have been published, developed, and endorsed. The published ACUITy recommendations state when to order a chest radiograph (2021), when not to order a CT/MRI scan for a headache (2022), and when to order an MRI scan for low back pain (2020) (18).

How then, do we assimilate these guidelines into AI models to avoid over-utilisation? In Singapore, as a behavioural cue for ordering clinicians to adhere to best practice standards, MOH and Singhealth in Singapore worked together to hardwire the “MRI for low back pain” ACG as a radiology order form into the electronic medical records (EMR) system. Similarly, to facilitate deployment in US and parts of Europe, appropriate use criteria have been commercially integrated as clinical decision support tools into EMR and order system. An example of this is the American College of Radiologist (ACR) Select® (19).

In another Singaporean example, Kedang Kerbau Women's and Children's Hospital (KKWCH) developed and implemented a deep learning model that enables automatic triaging of unstructured free-text paediatric MRI brain orders, in accordance with American College of Radiology (ACR) appropriate use criteria (20). This allows quicker and more appropriate disposition of patients to either ultrafast/abbreviated protocols or routine MRI brain, with the latter reserved for requests with higher probability of having brain abnormalities. Integrating evidence-based guidelines with machine learning can help to streamline the triaging process, allowing for more cost-effective use of limited, valuable resources (20).

### 4.2. Image acquisiton, processing and triage

AI shows tremendous promise in the field of MR imaging processing. SwiftMR™ is the flagship product of AIRS Medical, a healthcare AI startup based in Korea. By reconstructing high-quality images from a shorter, low-quality scan, SwiftMR™ promises to half the MRI scan time (21). The technology also seamlessly integrates into existing imaging infrastructure without the need for additional hardware, thus potentially reducing overhead costs while improving productivity within a short span of time. Without compromising on image quality, the shorter scan times can help healthcare institutions tackle their MRI backlog, potentially bringing forward diagnoses and thus treatment. In another example, with the help of AI and generative networks, investigators successfully outperformed state-of-the-art compressed sensing MRI reconstruction methods in both speed and image quality (22). The cost-effectiveness of such solutions is not to be underestimated, as they conceivably drive up revenue while at the same time reduce costs. SwiftMR™ expects its technology to increase the expected annual revenue of each MR scanner by USD$900,000 (23).

In the field of CT, shortening scan times may not have a similar outsized impact. Instead, AI can add value in reducing ionizing radiation via acquisition of sparse view images compared to conventional CT images while maintaining image quality (24, 25). Other major benefits AI solutions can bring include assisting with pre- and post-processing for CT image optimisation with the goal of reducing contrast media exposure, reducing need for repeat scans and improving picture quality (26). If these tasks can be automated or at least semi-automated, radiographer productivity can be improved.

Fluoroscopy remains an indispensable tool in diagnostic radiology, interventional radiology and endoscopic surgery. Bang et al. showed that an AI equipped fluoroscopy system for endoscopic surgery successfully reduced radiation exposure to patients as well as scatter effect to endoscopy personnel (27). In interventional radiology, 3D digital subtraction angiography images can be obtained from ultra-sparse 2D projections thus reducing radiation dose without compromising on fluoroscopic image quality (28).

In conventional radiography, the focus of AI solutions shifts to interpretation and worklist prioritisation. During the COVID-19 pandemic, a collaboration between Tan Tock Seng Hospital, Institute for Infocomm Research (I^2^R, A*Star) and Institute of High Performance Computing (IHPC, A*Star),, developed *RadiLogic*, a deep learning model that interprets chest radiographs quickly, prioritising abnormal radiographs for early review by the radiologist (12). The team successfully deployed the solution in a real-world clinical setting and effected a 22% reduction in turnaround times (12). Such AI tools not only improve productivity of the radiologists but also streamline the clinical workflow by prioritising sick patients that require quicker disposition, especially in the setting of an infectious disease.

Pattern recognition without human bias and human error is a key advantage of AI technologies. This can manifest as models identifying the design of a failed total hip replacement implant pre-operatively, thus saving time and reducing overall healthcare costs (29) or as models providing real-time automatic prediction of treatment response to transcatheter arterial chemoembolization for hepatocellular carcinoma (30).

### 4.3. Image interpretation and reporting

In a recent article, we reviewed the AI technologies for prostate MRI interpretation as well as automated organ and lesion depiction for the purpose of co-registering across modalities to enhance and personalise diagnostic and treatment methods based on clinical risk of malignancy (31). AI successfully reduces the workload of radiologists and urologists via (i) automatic delineation of suspected cancer regions, (ii) planning of biopsy by aligning MRI and ultrasound images, and (iii) guiding real time ablation. Similar applications using convolutional neural networks to detect brain metastases using T1 MRI have been reported (32).

AI optimisation of radiology reports comes in the form of natural language processing (NLP) algorithms. Efforts to convert unstructured free-form text into structured reports have been promising (33) and is seen as a leap of improvement from voice recognition software.

As healthcare moves towards evidence-based algorithms for disease management, it becomes crucial for referring physicians to have quick and efficient access to actionable radiological reports (34). The use of structured reporting templates makes it easier for readers to comprehend the information and helps radiologists in including all relevant details without any omissions, while presenting the information in a consistent and organized manner.

The Asian Oceanian Society of Radiology is currently undertaking a survey at the regional level to assess the knowledge, attitudes, and practices of radiologists and referring clinicians. This is the first step towards promoting widespread adoption of structured radiology reports. In the same vein, Radiological Society of North America (RSNA) has amassed more than four hundred report examples of best practices for diagnostic reporting (35).

The advent of ChatGPT has brought the world's attention to large language models. With a written or dictated report accompanying almost every radiographic study, language processing models are inevitable in the field of radiology. Apart from the aforementioned, NLP models and structured reporting provide the opportunity for extensive data mining for creating AI and predictive algorithms (36). Furthermore, these language models can provide a framework for efficient audit via auto-annotation and report classification (37).

## 5. Precision care in population health

Screening is a cornerstone of Population Health, as preventive and participatory health takes centre-stage (5). The myriad use cases AI has in oncologic screening and imaging are well-documented (38), it is the opportunistic screening of major chronic diseases that bears further discussion.

Opportunistic screening refers to the detection of abnormal findings unrelated to the primary indication for imaging studies (e.g., detecting an adrenal nodule in a CT scan done for appendicitis). In the case of population, preventive and participatory health, this incidental imaging data can be used for purpose of wellness, prevention, risk profiling or presymptomatic detection of relevant disease. For example, in the field of osteoporosis and fragility fractures, both CT and MR-based AI algorithms have been shown to be useful in deriving bone mineral density (39, 40), helping to identify patients who are at risk and in turn reduce the burden that frailty has on the healthcare system. Automated CT-based algorithms analysing vertebral trabecular HU match current clinical reference standard FRAX for predicting risk of future osteoporotic fractures (41). Furthermore, FRAX is cumbersome and requires manual entry of a dozen data points.

In the field of metabolic syndrome and cardiovascular disease, qualitative and quantitative analysis of body composition and adipose tissue depots using MR and CT have shown promise (42–45). Automated quantitative tissue biomarkers derived from CT scans can outperform established clinical parameters (Framingham risk score and BMI) for pre-symptomatic risk stratification for future serious adverse events and for predicting metabolic syndrome (42, 46). These tools can potentially allow for early detection of patients at risk, triggering early treatment protocols and thus reduce the overall chronic disease burden. These are especially important in any modern society grappling with an ageing population.

## 6. Democratization of radiology expertise through AI

AI can enable democratization of healthcare expertise to the population, thereby enhancing personalized care (47–49). However, we opine that the niche domain of radiology should reside primarily within the provider space. Instead, radiologist skill sets and insights derived from imaging examinations can be incorporated into AI algorithms and democratized to other healthcare practitioners. This is perhaps best illustrated in a hypothetical example (50): a patient with history of heart failure and on fluid restriction consults with a primary care clinic for breathlessness. He undergoes a chest radiograph and quickly gets up-triaged because the polyclinic's built-in AI algorithm (trained, monitored and audited by radiologists) diagnoses pulmonary congestion, pleural effusions and cardiomegaly. The attending physician rightfully refers him to the emergency department expeditiously where he gets the intravenous diuresis he requires. All this can happen just as a radiologist's report is churned out.

From the example above, we see that we would not only address shortages in radiologist manpower, but also empower non-radiologists to utilize radiology findings for clinical decision-making at the point of care. In the context of Population Health, the value proposition is strongest for physicians at the front-line, notably primary and community care providers. Here is where timely assessment and right-siting of care will see expedited treatment for patients requiring care in tertiary facilities, and continuation of care for lower acuity patients within the community setting, thereby lowering overall cost of care provision.

## Conclusion

In summary, AI can significantly transform the practice of Radiology, by improving productivity of the radiology workforce, while creating new opportunities to better support precision care in the global shift towards Population Health.
